# Legacy (Organochlorine) and Current Use (Neonicotinoid) Pesticides in Fish from Two Subtropical River Basins

**DOI:** 10.1007/s00244-026-01219-z

**Published:** 2026-10-26

**Authors:** Marelize Marsay, Chinemerem R. Ohoro, Collins Nimako, Yared B. Yohannes, Yoshinori Ikenaka, Nico J. Smit, Mayumi Ishizuka, Victor Wepener

**Affiliations:** 1 Water Research Group, Unit for Environmental Sciences and Management, North-West University, Potchefstroom, South Africa; 2 Department of Environmental Science, School of Ecological and Human Sustainability, College of Agriculture and Environmental Sciences, University of South Africa, Gauteng, South Africa; 3 Laboratory of Toxicology, Department of Environmental Veterinary Sciences, Faculty of Veterinary Medicine, Hokkaido University, Sapporo, Japan

## Abstract

The rapid population growth across Africa has increased the demand for food production, resulting in increased pesticide use. South Africa is among the top four pesticide importers in Sub-Saharan Africa, raising significant concerns regarding the contamination of freshwater ecosystems. Organochlorine pesticides (OCPs), historically used for agriculture and vector control, persist in the environment due to their high lipophilicity and long half-lives. Despite bans on many OCPs, residues remain in aquatic systems, potentially affecting non-target organisms such as fish. Similarly, neonicotinoids, introduced as safer alternatives, are now linked to adverse effects on aquatic biota, yet little is known about their bioaccumulation in wild fish species in southern Africa. This study investigated 13 neonicotinoids and 19 OCPs in fish muscle tissue from the Inkomati and Letaba catchments. Only two OCPs, *p,p’*-DDE and *p,p’*-DDD, were detected, likely due to ongoing DDT use in malaria control. Concentrations were generally low and varied between sites, with higher values found in the Letaba catchment. Neonicotinoids were detected at low concentrations, with acetamiprid and imidacloprid being the most prevalent, although the concentrations were lower than those reported globally. Muscle tissue, representing long-term exposure, may underestimate total pesticide burdens due to lower lipid content than other tissue types. Findings suggest that the detected residues reflect the persistent legacy of historical DDT use, potential ongoing localised DDT use for malaria vector control, as well as ongoing neonicotinoid use in agricultural systems. Although concentrations were below levels of concern for vertebrates, this study highlights the need for continued monitoring and expanded tissue analysis to better understand bioaccumulation and ecological risks in southern African freshwater systems.

**Supplementary Information:**

The online version contains supplementary material available at https://doi.org/10.1007/s00244-026-01219-z.

## Introduction

To keep up with the growing population in Africa, there is an increase in pesticide use for successful food production (Querner et al. 2016). South Africa is one of the four biggest importers of pesticides in Sub-Saharan Africa (Quinn et al. 2011). Although the use of many organochlorine pesticides has declined due to regulatory restrictions, legacy compounds have historically been used in South African agriculture and may persist in environmental matrices due to their chemical stability (Dabrowski 2022). Neonicotinoids have become increasingly important in South African crop production, with compounds such as imidacloprid and thiamethoxam widely applied as seed treatments and foliar sprays on crops (Dabrowski 2022). The continued presence of legacy pesticides and the increasing use of emerging pesticides such as neonicotinoids, continues to pose risks to aquatic ecosystems due to their persistence, bioaccumulation potential, and toxic effects on fish and other aquatic organisms (Uhl et al. 2015; Gerber et al. 2016; Klingehöfer et al. 2022).

Organochlorine pesticides (OCPs) are persistent organic pollutants that were designed to interfere with the physiological functioning of target organisms by opening the sodium channels in neurons for extended periods, overstimulating the nervous system (Karami-Mohajeri and Abdollahi 2011). Countries in southern Africa have a long history of OCP usage for agriculture and disease vector control programs (Darko et al. 2008; Buah-Kwofie et al. 2018). Previous studies across Africa have reported concentrations ranging from below detection limits to approximately 290 μg/L in water and several hundred μg/kg in sediments, reflecting the persistence of these compounds and their continued presence in aquatic environments despite restrictions and bans on many OCPs (Olisah et al. 2020). Their widespread occurrence, lipophilic nature and persistent nature in the environment promote their accumulation in the fatty tissues of aquatic organisms (Gerber et al. 2016; Olisah et al. 2020).

Some of these legacy pesticides used in South Africa include DDT, dieldrin, aldrin and chlordane, many of which have been banned worldwide due to their adverse effects on organisms (Ayele et al. 2022). When these pesticides came into circulation, there was little knowledge about the effect they would have on non-target organisms (Martyniuk et al. 2020). These OCPs are now known for causing harmful effects on aquatic ecosystems, and it is thus important to monitor their bioaccumulation in organisms to assess potential impacts (Gerber et al. 2016). Some of these harmful effects have been linked to endocrine disruption resulting from these compounds’ ability to interfere with thyroid hormone function and adversely affect neurobehavioural development and reproductive systems in both animals and humans (Olisah et al. 2020).

Neonicotinoids, a chemical pesticide modelled after nicotine, were introduced in the early 1990s, with imidacloprid being the first to market (Tišler et al. 2009). Neonicotinoids were initially considered as a potentially safer alternative to older insecticides such as organochlorines and organophosphates. Neonicotinoids are synthetic insecticides that act as agonists of nicotinic acetylcholine receptors (nAChRs), causing prolonged stimulation of the nervous system and subsequent neurotoxicity. Their generally high water solubility and systemic properties facilitate their transport into aquatic environments, where they can persist and where they are known to have adverse effects on non-target organisms. Although they have a selective affinity for insect nAChRs, exposure can disrupt neural, behavioural and physiological processes in non-target organisms such as birds, mammals, aquatic life, and bees (Uhl et al. 2015; Orikpete et al. 2025). Commercially available neonicotinoids are acetamiprid, clothianidin, dinotefuran, imidacloprid, nitenpyram, thiacloprid and thiamethoxam (Malhotra et al. 2021).

Neonicotinoids are extensively used in agriculture in South Africa, particularly in crops such as maize, cotton, rape-seed oil, sunflower, and sugarcane (Dabrowski 2022). However, their high leaching potential is concerning since this can lead to the contamination of nearby water sources, posing hazards to aquatic life (Iturburu et al. 2017; Malhotra et al. 2021). Neonicotinoids have been detected in African surface waters and sediments at concentrations ranging from ng/L levels to several mg/L, depending on the compound, location, and environmental matrix. Reported experimental aquatic toxicity concentrations ranged from 0.30 to 130 mg/L in aquatic organisms (Orikpete et al. 2025). Exposure to these neonicotinoids has also been associated with neurotoxicity, endocrine disruption, altered gene expression, and reduced biodiversity (Marsay et al. 2025; Orikpete et al. 2025).

The Inkomati and Letaba catchments are among the most intensively utilised river systems in southern Africa and are subject to substantial agricultural pressures. Both catchments support extensive irrigated agriculture, including sugarcane, citrus, subtropical fruit, cotton, and maize production, which rely heavily on surface water abstraction and have the potential to contribute pesticide residues to adjacent aquatic ecosystems (Dabrowski et al. 2015; Dabrowski 2022). The Inkomati River is a transboundary system shared by South Africa, Eswatini, and Mozambique, where water resources are heavily committed to agricultural, domestic, and industrial demands (Kahinda et al. 2016; Erasmus et al. 2023). The Letaba catchment forms part of the broader Limpopo River basin, where irrigation constitutes the dominant water use (Gallego-Ayala and Juízo 2014). Importantly, the Letaba River flows through the Kruger National Park before its confluence with the Olifants River, making it particularly important to understand upstream contaminant sources and potential pesticide inputs that may affect this internationally significant conservation area and its aquatic ecosystems.

The combination of intensive agricultural activity, high water demands, and transboundary water movement makes the Inkomati and Letaba catchments particularly relevant for investigating the occurrence and accumulation of legacy and emerging pesticides in freshwater ecosystems. Previous pesticide use maps for South Africa have also identified these regions as areas of extensive agricultural activity and potential pesticide application (Dabrowski et al. 2015), further supporting their selection for investigating pesticide residues in freshwater fish. Fish have been reported to accumulate higher pesticide concentrations than the surrounding water, while sediments act as a sink for persistent contaminants (Darko et al. 2008). Even though pesticides are used in high volumes in South Africa, there is limited information on the bioaccumulation of OCPs, and none on neonicotinoids in fish tissues. This study aimed to determine the occurrence and concentrations of neonicotinoids and organochlorine pesticides in fish muscle tissue from the Inkomati and Letaba catchments and to provide baseline information on pesticide residues in wild freshwater fish in southern Africa.

## Materials and Methods

### Study Sites

The eastern Letaba River Basin, one of the most important river basins in north-eastern South Africa supports intensive small- and large-scale commercial farming practices (Dickens et al. 2023). Tzaneen Dam (23.8142°S, 30.1436°E), the second largest dam in the catchment, serves as the primary freshwater source for domestic use and plays a crucial role in sustaining the natural flow of river further downstream, which includes South Africa’s largest conservation area, the Kruger National Park (KNP). The section between the Tzaneen Dam, Letaba River (23.6491°S, 30.6586°E), and the KNP is the most ecologically modified section of the basin due to upstream impoundments, large weirs and the main problem linked with intensive irrigated agriculture (fertilizers, salts and pesticide runoff). Agriculture activities in the area include livestock rearing (cattle and game) and diverse crop production, including citrus, bananas, mangoes, avocados, nuts and tomatoes (Querner et al. 2016).

**Fig. 1:**
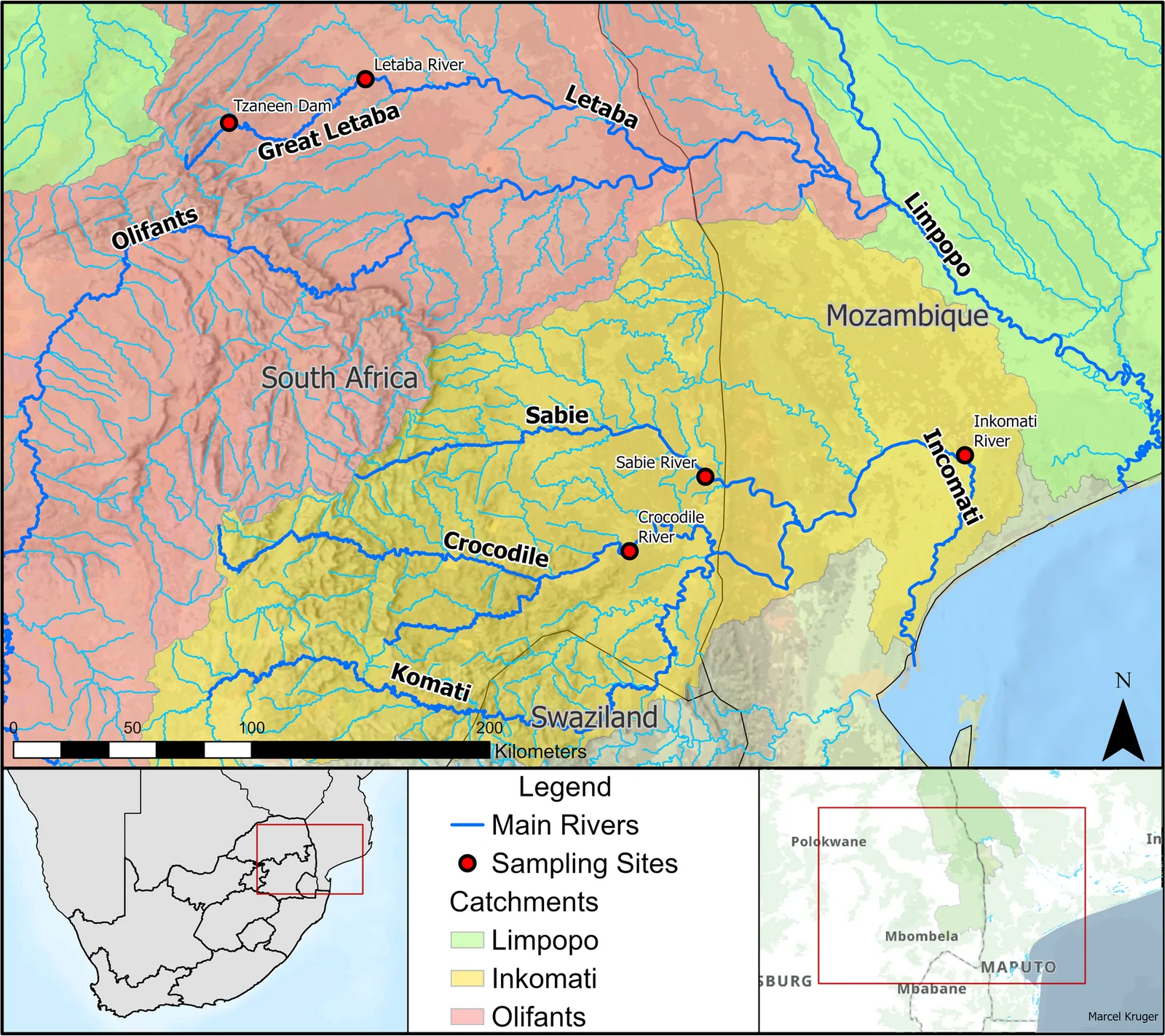
Map of sampling sites in the Letaba, Sabie, Crocodile, and Inkomati rivers, and Tzaneen Dam

The Inkomati River Basin spans through eastern South Africa, Eswatini, southern Mozambique and into the Indian Ocean (Erasmus et al. 2023). The Crocodile River (25.43077°S, 31.65537°E) is the main tributary of the Komati River and joins just before the Mozambique border. The Sabie River (25.14995°S, 31.94141°E), which flows through the Kruger National Park, then joins the Komati River and forms the Inkomati River (25.0689°S, 32.92141°E) in Mozambique (Fig. 1). Along its upper reaches, the river flows through regions subjected to industrial and agricultural activities, large-scale silviculture, and wastewater treatment facilities. The lower reaches in Mozambique are primarily influenced by agriculture, particularly sugarcane cultivation (Gallego-Ayala and Juízo 2014).

### Sample Collection and Preparation

Fish were sampled from the five sites using cast and fyke nets, rod and reel, baited longlines, and electrofishing. In situ water quality parameters [pH, electrical conductivity (EC), temperature, dissolved oxygen (DO)] were measured at each site with handheld meters (ExStik II EC500 and ExStik II DO600, Extech Instruments)(Online Resources Table S6). Water temperature was relatively consistent, ranging from 20.7 ± 0.10 to 22.4 ± 1.35 °C, while dissolved oxygen ranged from 6.9 ± 0.73 to 9.7 ± 0.15 mg/L. pH ranged from 7.5 ± 0.09 to 8.6 ± 0.14, whereas electrical conductivity showed greater spatial variation, increasing from 58 ± 27.4 µS/cm at Tzaneen Dam to 1162 ± 32.3 µS/cm at the Inkomati River.

In total 12 giant mottled eels—*Anguilla marmorata* (Inkomati River), seven longfin eels—*Anguilla mossambica* (Inkomati River), 15 African sharptooth catfish—*Clarias gariepinus* (Crocodile, Sabie and Inkomati rivers, Tzaneen Dam), five redbreast tilapia—*Coptodon rendalli* (Letaba River), 19 tigerfish—*Hydrocynus vittatus* (Crocodile, Sabie and Inkomati rivers), 17 largescale yellowfish—*Labeobarbus marequensis* (Letaba River), one red-eye labeo—*Labeo cylindricus* (Letaba River), five Limpopo bulldog—*Marcusenius krameri* (Letaba River and Tzaneen Dam) and one—Eastern butter catfish—*Schilbe depressirostris* (Tzaneen Dam) were collected in June 2022 and May 2023. Sampling effort was planned to target approximately 15 individuals per species per site. However, the occurrence and availability of target species varied among sampling sites. Consequently, some species-site combinations yielded substantially fewer individuals, with some represented by only one or two fish. These constraints were considered when interpreting the data, as low replication reduces statistical power and limits the strength of species- and site-specific comparisons.

Following capture, the specimens were identified using the appropriate taxonomic keys (Skelton 2024) and humanely killed using percussive stunning followed by spinal severance and pithing. Total length and mass were recorded for each specimen (Online Resources Table S7). Subsequently, dissected and muscle tissue was collected, stored in pre-cleaned polypropylene tubes and frozen at − 20 ºC until further analysis. Ethical approval for this project was received from the North-West University AnimCare animals research ethics committee (NWU-00267-17-A5, NWU-00781-22-A5) and the University of Mpumalanga animal ethics committee (AS/UMP/20,220,115). Permits for the collection of fish for research were obtained from South African National Parks (SS311), Ministry of Land and Environment, Mozambique (C2021/2022 − 00512), Mpumalanga Tourism and Parks Agency (MPB 5790 DD), the Department of Economic Development, Environment and Tourism, Limpopo, South Africa (ZA/LP/116078). Permission to access Tzaneen Dam was obtained from the Department Water and Sanitation, Limpopo, South Africa.

## Chemical Analysis

### Reagents and Standards

The targeted OCPs include α-BHC, β-BHC, γ-BHC (Lindane), δ-BHC, hexachlorobenzene, heptachlor, heptachlor-exo-epoxide, oxy-chlordane, trans-chlordane, cis-chlordane, trans-nonachlor, cis-nonachlor, aldrin, endrin, dieldrin, *p,pʹ*-DDE, *o,pʹ*-DDE, *o,pʹ*-DDD, *p,pʹ*-DDD, *o,pʹ*-DDT, and *p,pʹ*-DDT. The OCP-mix was obtained from Dr. Ehrenstorfer GmbH (Augsburg, Germany). Surrogate internal standard 3,3’,4,4’-tetrachlorobiphenyl (PCB 77) was purchased from Sigma-Aldrich (Tokyo, Japan). The targeted neonicotinoids include 4 OH-imidacloprid, 5 OH- imidacloprid, dinotefuran, acetamiprid, dm-acetamiprid, clothianidin, dm-clothianidin, imidacloprid, nitenpyram, thiacloprid, thiamethoxam, sulfoxaflor, and were obtained from Wako Pure Chemical Co. (Osaka, Japan). The neonicotinoid internal standards: thiamethoxam-d4, thiacloprid-d4, nitenpyram-d3, sulfoxaflor13C-2-D3-15N2, imidacloprid-D4, dm-clothianidin-13C-15N2, dm-acetamiprid-D3, dinotefuran-D3, clothianidin-D3, acetamiprid-D3, 4 OH-imidacloprid-13C-15N2, and 5 OH-imidacloprid-13C-15N2 were obtained from Hayashi Pure Chemical Industry, Ltd. (Osaka, Japan). All solvents and reagents used were analytical grade and purchased from Wako Pure Chemical Co. (Osaka, Japan) and Kanto Chemical Co., Inc. (Tokyo, Japan).

### Organochlorine Extraction and Analysis

Freeze-dried samples were homogenised using a mortar and pestle and approximately 0.2 to 1.4 g of the homogenised sample was weighed into 15 mL centrifuge tube and spiked with 50 µL (1 ppm) PCB 77 as an internal standard and allowed to equilibrate for 2 h. Using a mortar and pestle was a suitable homogenising the relatively larger tissue samples. The extraction protocol was conducted at room temperature unless otherwise stated. Whereafter, 5 mL of 0.1% formic acid in acetonitrile was added, capped and shaken. The mixture was ultrasonicated for 5 min and shaken with a mechanical shaker (Yamato Scientific, Japan) at maximum speed for 10 min. This mixture was centrifuged (Thermofisher Scientific, Germany) for 5 min at 10,000 rpm, after which the supernatant was transferred to an EMR-lipid tube (5982–1010, Agilent, USA), which had been preconditioned with 5 mL of distilled water. After the addition of the sample, the tube was vortexed immediately for 1 min and then placed on a mechanical shaker for 10 min, followed by a second centrifugation at 10,000 rpm for 5 min. The supernatant was then transferred into a 15 mL tube containing 1.4 g of MgSO_4_ and 0.6 g of NaCl (Agilent, USA), vortexed for 1 min, and centrifuged at 10,000 rpm for 5 min. Finally, 1 mL of the supernatant was transferred to a GC-vial for analysis.

For OCPs, analytes were quantified using a GC-2030 coupled with a triple quadrupole mass spectrometer (GCMS-TQ8050NX, Shimadzu). Chromatographic separation was carried out using an Rxi-5Sil MS capillary column (30 m × 0.25 mm × 0.25 μm, Restek, USA). Samples were injected at a volume of 1 µL at an inlet temperature of 250 °C using splitless mode. Helium (≥ 99.999%) was used as the carrier gas with linear velocity in flow control mode, 100.1 kPa pressure, 30.0 mL/min total flow rate, 1.69 mL/min column flow, 47.2 cm/s linear velocity, and 5 mL/min purge flow. The oven temperature program started at 50 °C for 1 min, increased to 200 °C at 30 °C/min, and finally ramped to 260 °C at 5 °C/min, where it was held for 3 min. The total program duration was 20 min. The mass spectrometer system was operated in the electron ionization (EI) mode at 70 eV. The ion source and interface temperatures were 230 and 250 °C, respectively.

### Neonicotinoid Extraction and Analysis

Approximately 0.1 to 0.6 g of freeze-dried tissue sample was weighed and placed into a 1.5 mL Eppendorf tube, spiked with 100 µL (5 ppb) neonicotinoid internal standard master mix and then vortexed (Vortex-Genie 2, USA) for 30 s. The sample was then allowed to stand for 5 min. The extraction protocol was conducted at room temperature unless otherwise stated. Subsequently, 500 µL of 0.1% formic acid in acetonitrile and three pieces of Zirconia beads (2 mm. ø) were added, and the sample was homogenized for 5 min using a tissue lyser (Retsch, Germany). Given the smaller tissue mass used for the extraction, samples were homogenised using a tissue lyser with Zirconia bead to facilitate efficient tissue disruption and mixing within the small-volume tube. The mixture was further centrifuged at 10,000 rpm for 5 min. The supernatant was collected and dispensed into a 5 mL Eppendorf tube, and another 500 µL of 0.1% formic acid in acetonitrile was added to the residue. The mixture was vortexed for 3 min and centrifuged at 10,000 rpm for 5 min. This supernatant was collected and added to the previous.

The supernatant (total volume of 1 mL) was diluted with 3 mL 0.3% acetonitrile in distilled water and then purified by solid phase extraction using InertSep C18 SPE cartridges (200 mg/ 3 mL, GL Sciences Inc., Japan) that had been preconditioned with 1 mL acetonitrile. The sample was then loaded onto the cartridge and eluted gently at a flow rate of 1 mL/min into a 15 mL centrifuge tube. The cartridge was then washed with 1 mL 0.3% acetonitrile in distilled water and added to the first eluate. Another 3 mL of 0.1% formic acid in acetonitrile was added, vortexed for 3 min, whereafter 1 g MgSO_4_ was added and shaken immediately. The sample was then centrifuged at 10,000 rpm for 10 min. The upper organic phase was collected into a glass test tube and evaporated to dryness under vacuum using a centrifugal evaporator (CVE−220D with UT-2000, EYELA, Japan). The sample was reconstituted with 200 µL 20% methanol, vortexed, and ultrasonicated for 3 min. Finally, the 200 µL reconstituted sample was transferred to a GC-vial for analysis.

A Kinetex® 2.6 µm Binephenyl 100 Å (100 mm × 30 mm) column (Showa Denko) was fitted with the Shimadzu 20 A series liquid chromatography-electrospray ionization-tandem mass spectrometry (LC-ESI/MS/MS) equipment. Solvents A and B were applied with the following gradient: t = 0 to 2 min, 20% solvent B; t = 11 min, 95% solvent B; t = 11 to 13 min, 95% solvent B. Solvents A and B consisted of 0.1% (v/v) formic acid and 10 mM ammonium acetate in water and methanol, respectively. The flow rate was 0.4 mL/ min, and the column oven temperature was 45 °C.

### Quality Assurance and Control

In the current study, the internal standard (IS) method was used to quantify the neonicotinoids and OCPs in the samples. Seven-point standard calibration concentrations ranging from 1 to 100 ppb were used to establish the calibration curve for the OCPs. Calibration curves were plotted using the ratio between the standard peak area and IS peak area. Regression analyses were performed on the calibration curves (see Online Resource Tables S1 and S2). Background contamination was monitored by analyzing blanks along with the samples; any compounds detected in the procedural blanks were subtracted from the samples. A laboratory control sample spiked with a concentration of 5 ppb was analysed with every batch of 24 samples. These samples were extracted, purified and analysed in the same manner as the fish tissues, with the addition of internal standards to account for any compounds lost during the extraction process. The recovery rates for samples spiked with 5 ppb ranged between 72 and 140%, which is within an acceptable range (see Online Resource Table S3). β and γ-BHC were grouped and referred to as Lindane, while *o,p’*-DDT and *p,p’*-DDD, referred to as *p,p’*-DDD, since these peaks could not be distinguished from one another after analysis.

For neonicotinoids, 11-point standard calibration concentrations ranging from 0.05 ppb to 20 ppb were used to establish the calibration curve (see Online Resource Table S4). Blank samples and two laboratory control samples spiked with low (1 ppb) and high (10 ppb) concentrations were analysed with every batch of nine samples. High recovery rates (76 to 125%) were found at the low spiked control sample (1 ppb), except for 4OH-Imidacloprid (24%), 5OH-Imidacloprid (15%) and Thiacloprid (46%), which had very low recovery rates indicating that the extraction protocol was not effective for these three compounds. Lower recovery rates (37 to 62%) were found at the high spiked control sample (10 ppb) (see Online Resource Table S5). The lower recovery rate percentages likely attributable to a combination of factors, which can can be due to the concentration solvent ratio used during the extraction protocol, where a higher solvent volume used could have possibly increased the recovery rates and increased analyte losses during sample preparation (freeze drying tissue samples) and clean-up procedures. Consequently, the concentrations reported for these compounds should be interpreted cautiously, and the lower recoveries are recognised as a limitation of the analytical method. The non-detection of these compounds cannot be interpreted as definitive evidence of their absence. However, the concentrations expected in the fish tissue were expected to be closer to the lower concentration range presented by the 1 ppb spike level, the recovery rates obtained at this concentration were considered more relevant to the expected sample concentrations and were therefore considered adequate for the purposes of this study.

### Data Analysis

The limit of detection (LOD) and limit of quantification (LOQ) were determined using a regression analysis of the calibration curves, where LOD = 3(Sb/b) and LOQ = 9(Sb/b) with Sb = slope uncertainty and b = slope (Schoeman et al. 2015). Samples were analysed in different batches, each with a specific LOD due to instrument variation (see Online Resource Tables S1, S2 and S4). For descriptive and statistical analysis, values below the detection limit were substituted using half the LOD specific to each sample’s analytical batch. For descriptive statistics (Tables 1 and 2), mean concentrations were calculated using both detected and non-detected samples. In Tables 1 and 2 concentrations below the analytical LOD were still assigned a value of LOD/2 to avoid overestimating mean concentrations by excluding non-detects. The concentration data presented in Tables 1 and 2 are also provided as bar graphs in the Online Resources (Figs. S1–S5) to facilitate visual comparison among fish species and sampling sites.

**Table 1:** OCP concentrations measured in the muscle tissue of fish collected from the Inkomati and Letaba catchments (ng/g dry weight, mean ± standard error = mean concentrations were calculated by including non-detects, which were assigned a value of LOD/2, <LOD = below detection limit, n = number of fish which had concentrations above the detection limit)

Fish species	Site	*p,p’*-DDE			*p,p’*-DDD		
		mean	min–max	n	mean	min–max	n
*Anguilla marmorata*	Inkomati	3.1 ± 0.7	<LOD–9.51	2/12	4.2 ± 0.9	<LOD–12.17	1/12
*Anguilla mossambica*	Inkomati	<LOD	<LOD	0/7	<LOD	<LOD	0/7
*Clarias gariepinus*	Crocodile	<LOD	<LOD	0/4	<LOD	<LOD	0/4
	Sabie	<LOD	<LOD	0/4	<LOD	<LOD	0/4
	Inkomati	11.1 ± 7.3	<LOD–32.91	1/4	1.9 ± 1.3	<LOD–5.784	2/4
	Letaba (Tzaneen Dam)	446.9 ± 416.6	<LOD–1280	1/3	16.1 ± 12.5	<LOD–41.13	1/3
*Coptodon rendalli*	Letaba	<LOD	<LOD	0/5	<LOD	<LOD	0/5
*Hydrocynus vittatus*	Crocodile	6.9 ± 5.4	<LOD–55.95	1/10	5.9 ± 1.3	<LOD–17.52	1/10
	Sabie	<LOD	<LOD	0/5	<LOD	<LOD	0/5
	Inkomati	<LOD	<LOD	0/4	<LOD	<LOD	0/4
*Labeobarbus marequensis*	Letaba	47.2 ± 8.3	<LOD–110.4	13/17	<LOD	<LOD	0/17
*Labeo cylindricus*	Letaba	<LOD	<LOD	0/1	<LOD	<LOD	0/1
*Marcusenius krameri*	Letaba	185.4 ± 175.9	<LOD–361.3	1/2	21.9 ± 18.4	<LOD–40.32	1/2
	Letaba (Tzaneen Dam)	52.0 ± 42.5	<LOD–137	1/3	11.2 ± 3.8	<LOD–18.91	1/3
*Schilbe depressirostris*	Letaba (Tzaneen Dam)	51.6	51.6	1/1	<LOD	<LOD	0/1

**Table 2:** Neonicotinoid concentrations measured in the muscle tissue of fish collected from the Inkomati and Letaba catchments (pg/g dry weight, mean ± standard error = mean concentrations were calculated by including non-detects, which were assigned a value of LOD/2, <LOD = below detection limit, n = number of fish which had concentrations above the detection limit)

Fish species	Site	Acetamiprid			Dinotefuran			Imidacloprid			Clothianidin			Thiamethoxam		
		mean	min–max	n	mean	min–max	n	mean	min–max	n	mean	min–max	n	mean	min–max	n
*Anguilla marmorata*	Inkomati	4.9 ± 0.4	2.8–7.0	12/12	27.9 ± 9.7	<LOD–134.8	1/12	1476.0 ± 277.7	642.6–3558	12/12	146.2 ± 59.1	<LOD–785.7	2/12	19.7 ± 11.3	<LOD—143.8	1/12
*Anguilla mossambica*	Inkomati	4.6 ± 0.9	<LOD–8.7	6/7	<LOD	<LOD	0/7	1252.0 ± 533.4	<LOD–4191.0	6/7	181.4 ± 66.7	<LOD–552.6	3/7	<LOD	<LOD	0/7
*Coptodon rendalli*	Letaba	13.9 ± 3.8	<LOD–21.0	3/5	<LOD	<LOD	0/5	<LOD	<LOD	0/5	<LOD	<LOD	0/5	<LOD	<LOD	0/5
*Clarias gariepinus*	Crocodile	6.7 ± 1.2	4.5–9.9	4/4	<LOD	<LOD	0/4	2118.3 ± 1542.0	168.4–6715.7	4/4	<LOD	<LOD	0/4	<LOD	<LOD	0/4
	Sabie	6.7 ± 2.2	2.2–12.6	4/4	<LOD	<LOD	0/4	475.6 ± 110.3	247.4–765.9	4/4	<LOD	<LOD	0/4	<LOD	<LOD	0/4
	Inkomati	10.0 ± 4.1	3.4–22.1	4/4	126.0 ± 64	<LOD–270.1	2/4	926.9 ± 543.9	<LOD – 2518.2	3/4	<LOD	<LOD	0/4	11.2 ± 2.7	<LOD—19.3	1/4
	Letaba (Tzaneen Dam)	7.6 ± 2.2	<LOD–12.0	2/3	<LOD	<LOD	0/3	<LOD	<LOD	0/3	<LOD	<LOD	0/3	<LOD	<LOD	0/3
*Hydrocynus vittatus*	Crocodile	4.5 ± 0.9	3.0–13.0	10/10	<LOD	<LOD	0/10	454.2 ± 318.0	<LOD–3303.1	6/10	<LOD	<LOD	0/10	<LOD	<LOD	0/10
	Sabie	3.0 ± 0.6	<LOD–4.67	4/5	<LOD	<LOD	0/5	265.3 ± 73.4	<LOD–416.9	4/5	<LOD	<LOD	0/5	<LOD	<LOD	0/5
	Inkomati	4.4 ± 0.8	3.4–6.6	4/4	<LOD	<LOD	0/4	117.4 ± 44.3	<LOD–244.1	2/4	<LOD	<LOD	0/4	<LOD	<LOD	0/4
*Labeobarbus marequensis*	Letaba	17.4 ± 1.9	<LOD–38.2	15/17	<LOD	<LOD	0/17	<LOD	<LOD	0/17	<LOD	<LOD	0/17	<LOD	<LOD	0/17
*Labeo cylindricus*	Letaba	<LOD	<LOD	0/1	<LOD	<LOD	0/1	<LOD	<LOD	0/1	<LOD	<LOD	0/1	<LOD	<LOD	0/1
*Marcusenius krameri*	Letaba	14.1 ± 0.4	13.7–14.5	2/2	<LOD	<LOD	0/2	<LOD	<LOD	0/2	<LOD	<LOD	0/2	<LOD	<LOD	0/2
	Letaba (Tzaneen Dam)	16.4 ± 1.1	14.2–18.0	3/3	<LOD	<LOD	0/3	<LOD	<LOD	0/3	<LOD	<LOD	0/3	<LOD	<LOD	0/3
*Schilbe depressirostris*	Letaba (Tzaneen Dam)	19.6	19.6	1/1	<LOD	<LOD	0/1	<LOD	<LOD	0/1	<LOD	<LOD	0/1	<LOD	<LOD	0/1

Statistical analysis was performed using GraphPad Prism 11. For each pesticide, concentrations were compared among sampling sites within the same fish species, as well as among different fish species at the same sampling site. Where all individuals of a particular fish species at a specific sampling site had pesticide concentrations below the analytical detection limit, that species-site combination was excluded from statistical analyses. Significance was set at p ≤ 0.05. Data were tested for normality before statistical analysis. The Shapiro–Wilk and Kolmogorov–Smirnov normality tests were performed to confirm the normal distribution of the datasets. For datasets that met the assumption of normality, homogeneity of variance was assessed using the Brown-Forsythe test.

For the OCPs, a nonparametric Kruskal–Wallis test followed by Dunn’s multi-comparison test was performed since the dataset was not normally distributed. For neonicotinoid datasets that met the assumptions of normality and homogeneity of variance, differences among groups were assessed using a one-way analysis of variance (ANOVA), followed by Tukey's multiple comparisons test. For datasets that did not meet the assumptions, differences among groups were assessed using a Kruskal–Wallis test, followed by Dunn's multiple comparisons test. Only two fish species could be statistically compared in the Crocodile and Sabie rivers. For comparisons between two fish species, normally distributed datasets were analysed using Welch's t-test to account for unequal variances. Non-normally distributed datasets were analysed using the non-parametric Mann–Whitney U test.

## Results

### Organochlorine Pesticides

Of the 19 OCPs analysed in the fish tissue, only two were above the detection limit, i.e. *p,p’*-DDE and *p,p’*-DDD (Table 1). Of the 82 fish sampled, *p,p’*-DDE was detected in 21 fish from the Crocodile, Inkomati and Letaba Rivers, as well as Tzaneen Dam. While *p,p’*-DDD could only be detected in seven fish from the Inkomati and Crocodile Rivers, and Tzaneen Dam. There were no significant differences (p > 0.05) between sites or fish species. Overall, these two compounds were higher in the fish from the Letaba catchment when compared to those from the Inkomati catchment.

### Neonicotinoids

Of the 12 neonicotinoids analysed, only five were above the detection limit: acetamiprid, clothianidin, dinotefuran, imidacloprid and thiamethoxam (Table 2). Acetamiprid was the only compound detected at all sites, followed by imidacloprid, which was detected at the sites located in the Inkomati catchment (Crocodile, Inkomati and Sabie rivers). Clothianidin was found in only five of the 82 fish analysed, dinotefuran in only three and thiamethoxam in only two, all found in the Inkomati River. The only significant difference observed was for imidacloprid concentrations between *A. marmorata* and *H. vittatu*s in the Inkomati River. At the three sites (Crocodile, Inkomati and Sabie rivers) where both *H. vittatus* and *C. gariepinus* were collected *H. vittatus* contained lower concentrations than *C. gariepinus*, even though not always significant.

## Discussion

Beyond assessing human dietary exposure, fish muscle tissue serves as an indicator of contaminant bioaccumulation and internal exposure, providing insights into the bioavailability, uptake, and persistence of contaminants in aquatic ecosystems. Contaminant concentrations in muscle tissue also provide valuable baseline data for environmental monitoring by identifying contaminant occurrence, spatial patterns, and potential areas of concern within freshwater systems. The pesticides used on crops and for disease control infiltrate aquatic ecosystems and even though many of the OCPs are banned, they persist in the environment and are reflected in the organisms that are found in these systems. This study investigated the occurrence of legacy OCPs and emerging neonicotinoid insecticides in fish from the Letaba and Inkomati catchments. Overall, only a limited number of OCPs were detected in the fish tissue, with *p,p’*-DDE and *p,p’*-DDD being the only congeners present. Neonicotinoids were detected at very low concentrations and with low detection frequencies, suggesting limited bioaccumulation in fish. However, spatial differences between the catchments and species-specific ecological traits appear to influence contaminant exposure and accumulation.

### Organochlorine Pesticides

Out of the 19 OCPs analysed, only two were detected in the fish tissue, *p,p’*-DDE and *p,p’*-DDD. The detection of these two congeners may be due to the continued use of DDT in Limpopo, Mpumalanga, KwaZulu-Natal, where they are still used for indoor residual spraying (Buah-Kwofie et al. 2018; Pheiffer et al. 2018; Van den Berg et al. 2025). Countries in southern Africa continue to use DDT for indoor residual spraying programs, as there is no effective alternative to control vectors that transmit malaria since mosquitoes become resistant (Ayele et al. 2022). Once released into the environment, DDT can persist for long periods due to its transformation into the more stable metabolites *p,p’*-DDE and *p,p’*-DDD. Numerous studies have found that DDTs are the most abundant OCPs in freshwater ecosystems, with *p,p’*-DDE being the predominant and persistent congener (Barnhoorn et al. 2009; Gerber et al. 2016; Verhaert et al. 2017). This is likely due to the longer environmental half-lives of DDT and its metabolites compared to the other OCPs (Ayele et al. 2022).

Verhaert et al. (2017) reported lower ƩDDX concentrations in four species common to both studies (*C. gariepinus*, *H. vittatus*, *L. marequensis* and *S. depressirostris*) in the Olifants River, despite their sampling site being downstream of the sites investigated in the present study. In addition, DDT contributed to approximately 5% of the ƩDDX concentrations in their samples, whereas no parent DDT was detected in the present study. The predominance of *p,p'*-DDE and *p,p'*-DDD in the absence of detectable parent DDT suggests that the residues in fish tissue largely represent aged and degraded DDT residues that have persisted in the environment rather than substantial recent inputs of the parent compound. This is supported by the reported half-lives of these compounds in fish, with *p,p’*-DDT having a half-life of 8 months, whereas the half-life of *p,p’*-DDD and *p,p’*-DDE being approximately 7 years (Binelli and Provini 2003; Buah-Kwofie et al. 2018; Ayele et al. 2022). Trace concentrations of DDT below the method detection limit cannot be completely excluded; however, such low concentrations indicate no substantial or recent input of parent compound.

*Clarias gariepinus* from Tzaneen Dam had the highest concentration of *p,p’*-DDE, while *M. krameri* from the Letaba River had the highest *p,p’*-DDD concentration. Elevated concentrations were observed in benthic and omnivorous species that feed on sediment-associated organisms, such as the *C. gariepinus, L. marequensis*, M*. krameri*, and *S. depressirostris*. This suggests that species-specific ecological traits, such as habitat use, feeding behaviour, and dermal characteristics, together with sediment-associated pathways, may influence accumulation (Verhaert et al. 2017).

Variation in OCP concentrations in fish tissue may reflect differences in site contamination, however, variability among individuals at the same site made it difficult to compare the variation statistically. Overall, *p,p’*-DDE and *p,p’*-DDD concentrations were higher in the Letaba catchment than in the Inkomati catchment. Similarly, Heath and Claassen (1999) reported that fish from the Letaba River contained three orders of magnitude higher concentrations of these congeners than those measured in the Crocodile and Sabie rivers. This pattern was attributed to the extensive use of DDT for malaria vector control in areas surrounding the Letaba River during periods of an increase in malaria incidence. In a study by Gerber et al. (2015), 22 OCPs were analysed in the sediment from the Letaba River and only four were detected. In both low and high flow, trace amounts of *p,p’*-DDE and *p,p’*-DDD were present. These authors found that the OCP concentrations in the sediment were already lower than in previous studies and that the ƩDDT concentrations had decreased since 1998 (Naude et al. 1998; Gerber et al. 2015). This decrease in OCP concentrations was also reflected in the muscle tissue of tigerfish (*H. vittatus*) (Gerber et al. 2016). These trends suggest reduced inputs and a potential decrease in the use of DDT andsuggest that this historic use remains evident in the Letaba catchment, highlighting the persistence of DDT and its metabolites in the environment.

The *p,p’*-DDE concentrations in the current study were generally similar or lower when compared to those available in other southern African aquatic systems, with fish from the Letaba catchment generally being higher (Barnhoorn et al. 2009, 2015; Wepener et al. 2012; Buah-Kwofie et al. 2018; Pheiffer et al. 2018; Olisah et al. 2019) and similar to those found in studies performed in Uganda (4–15 ng/g) (Ssebugere et al. 2009) and Ethiopia (ranging 0.84 and 122.70 ng/g) (Deribe et al. 2013; Ayele et al. 2022). The *p,p’*-DDD concentrations measured in the Inkomati catchment were comparable to those reported for laboratory-depurated *C. gariepinus* (5 ± 2.6 ng/g) in Pheiffer et al. (2018). Overall, *p,p’*-DDD concentrations were similar to those reported in Ssebugere et al. (2009) in Uganda (2–16 ng/g), but were generally much lower than concentrations reported in studies conducted in South Africa and Ghana (Darko et al. 2008; Barnhoorn et al. 2009; Gerber et al. 2016; Buah-Kwofie et al. 2018; Pheiffer et al. 2018).

Comparisons between the present study and previously mentioned studies should be made with caution, as many studies report OCP concentrations on a wet weight or lipid weight basis, whereas concentrations in the present study are expressed on a dry weight basis. The removal of the water during freeze-drying reduces the mass of the sample and typically results in higher concentrations when expressed on a dry weight basis. Furthermore, lipid content was not quantified in the present study since the extraction protocol incorporated an EMR-Lipid clean up step to remove lipids from the sample extracts to minimise matrix effects during analysis.

### Neonicotinoids

As mentioned previously of the 12 neonicotinoids analysed, only five were detected in the fish tissue, namely acetamiprid, clothianidin, dinotefuran, imidacloprid and thiamethoxam. Information on neonicotinoid accumulation in fish remains limited internationally, particularly regarding their occurrence in wild freshwater fish. Available studies have largely focused on laboratory-based exposure experiments and toxicological effects, while field-based assessments of neonicotinoids in wild fish populations are scarce. Neonicotinoids are widely used to control insect pests affecting commercial crops, fruits, flowers, and decorative plants, and are applied either as seed dressings or foliar sprays (Zuščíková et al. 2023). Neonicotinoids can then enter aquatic ecosystems through processes such as agricultural and urban runoff (Dabrowski et al. 2015). Acetamiprid is among the most used neonicotinoid pesticides worldwide (Mutshekwa et al. 2023), which may explain why it was detected at all sampling sites and in almost all fish species in the present study. In both the Inkomati and Letaba catchments, the primary agricultural use of acetamiprid is citrus and cotton (Dabrowski 2022).

In a study by Ma et al. (2022), zebrafish (*Danio rerio*) were continuously exposed for 22 weeks to environmentally relevant concentrations of acetamiprid (0.15, 1.5, 15, 150 and 1,500 µg/L). At the end of the 22 weeks, acetamiprid concentrations within the fish exposed to 0.15 µg/L (the lowest concentration) were 140 ± 10 pg/g in females and 400 ± 30 pg/g for males. When compared to the acetamiprid concentrations found in the present study (< LOD to 38 pg/g) suggests either lower environmental acetamiprid exposure or limited accumulation within the muscle tissue. Yang et al. (2022) investigated the uptake, distribution, elimination and metabolism of clothianidin in zebrafish and found higher concentrations in the intestine and liver than in the muscle. The muscle tissue showed lower uptake and that it may have been detected in the muscle tissue from blood-tissue distribution. However, despite lower concentration in the muscle tissue, its large contribution to the total fish biomass may still pose a risk through the consumption of contaminated fish (Yang et al. 2022).

The most common neonicotinoid found together with acetamiprid is imidacloprid, although thiamethoxam and clothianidin are becoming increasingly prevalent. Imidacloprid is widely used in southern Africa on a variety of crops at relatively high concentrations in both the Inkomati and Letaba catchments (0.27–2.3 kg/ha) (Dabrowski et al. 2015). In the present study, however, it was only detected in fish from the Inkomati catchment. Pesticide use maps of South Africa indicate extensive imidacloprid application in the areas surrounding the Sabie and Crocodile rivers, which drain into the Inkomati River (Dabrowski et al. 2015). In contrast, although imidacloprid is also widely used in the Letaba catchment, its use is upstream of the sampling sites, and therefore, it was not detected in fish from this system. Imidacloprid is known to dissipate rapidly from the water column, primarily through photolysis and hydrolysis, with reported half-lives of only a few days depending on environmental conditions such as light intensity and temperature (Colombo et al. 2013; Rico et al. 2018; Marsay et al. 2025). This, together with adsorption to sediment, may further reduce its availability in the water column downstream and limit accumulation by aquatic organisms such as fish. Factors such as continuous inputs, physicochemical conditions, and microbial processes may also influence exposure duration. Field-based studies investigating imidacloprid residues and bioaccumulation in fish remain limited, highlighting the need for further research to better understand its environmental fate and accumulation potential.

Thiamethoxam is used on a wide variety of crops, while clothianidin is mainly applied as a seed dressing for cotton and maize in South Africa (Dabrowski 2022). Their relatively lower usage compared to imidacloprid may explain the absence of these compounds in fish from the South African sampling sites in the present study. Similarly to imidacloprid, thiamethoxam is not directly applied in the vicinity of the sampling sites in the Letaba catchment but is applied in the surrounding areas of the Crocodile River (Inkomati catchment) at much lower concentrations than imidacloprid (0.000092–0.0022 kg/ha) (Dabrowski et al. 2015). Clothianidin, dinotefuran, and thiamethoxam were only detected in fish from the Inkomati River in Mozambique, suggesting that the sources and usage patterns of the compounds may differ between the South African sampling sites and the downstream Mozambican portion of the catchment. The Crocodile River flows into Mozambique, where it joins the Komati River, which then becomes the Inkomati River. Until 2009, DDT was used for malaria vector control in Mozambique, after which there was a shift to pyrethroids and organophosphates and eventually the use of neonicotinoids (more specifically, clothianidin) until 2021 (Kiuru et al. 2025). Dinotefuran has also been reported for use in malaria vector control in Mozambique (Corbel et al. 2004). The occurrence of clothianidin and dinotefuran in fish from the Mozambican portion of the Inkomati River is therefore consistent with potential regional inputs from agricultural activities and vector control practices within the broader transboundary catchment.

Clothianidin was found exclusively in the two eel species, occurring in five of the 19 individuals. Dinotefuran was detected in two of the four sharptooth catfish and one of the 12 giant mottled eels, while thiamethoxam was detected in only two individuals overall (one sharptooth catfish and one giant mottled eel). Although these neonicotinoids were present in the fish tissue, they were detected at a low frequency, occurring in only a small proportion of the individuals analysed. The significantly higher imidacloprid concentrations observed in *A. marmorata* compared with *H. vittatus* at the Inkomati River. The species-specific ecological traits of eels and the sharptooth catfish may explain why only these species had detectable concentrations of all neonicotinoids at this site.

Sediment and organic matter can act as a persistent sink of pesticides, exposing benthic and sediment-associated fish long after their use has ceased or even when the use is intermittent (Gerber et al. 2015). These two species feed on a wide variety of organisms and are closely associated with sediment habitats, potentially increasing their exposure to sediment-associated pesticide residues. Their relatively high lipid content may also facilitate the partitioning and retention of lipophilic contaminants. It should be noted that both species are scaleless, which may increase their exposure through dermal uptake. Accumulation through the skin is an important route of exposure to fish, especially those that live in close contact with sediment and sediment-associated compounds (Nichols et al. 1996). A few of the very limited studies focusing on scale vs scaleless fish found that the presence of scales limited uptake of contaminants (Feng et al. 2019; Paul et al. 2025). These shared ecological characteristics may contribute to the detection of neonicotinoids in both species. However, differences in diet, habitat use, body composition and detoxification capacity may also contribute to differences in accumulation patterns.

Very few studies have investigated neonicotinoid residues in fish for African freshwater systems. Adegun et al. (2020) detected acetamiprid, thiacloprid and thiamethoxam in the muscle tissue of six fish species collected from the Owena River basin in Nigeria. Acetamiprid and thiamethoxam concentrations ranged between 10,000 to 160,000 pg/g, whereas no imidacloprid was detected. It is worth noting that Adegun et al. (2020) performed their extraction using wet fish tissue, whereas the present study used freeze-dried tissue. In contrast, a study of *Clarias anguillaris* and *Oreochromis niloticus* from two agricultural reservoirs in Ghana screened for 42 pesticides in the muscle tissue, and no neonicotinoids were detected (Adokiya et al. 2025). These limited and contrasting findings highlight both the scarcity of field-based data on neonicotinoids in African freshwater fish and the variability in their detection among aquatic systems. The generally low concentrations observed in the present study are consistent with the relatively low bioaccumulation potential of neonicotinoids compared to more hydrophobic legacy pesticides such as OCPs, although differences in environmental exposure, metabolism, tissue distribution and analytical sensitivity may also contribute to the observed concentrations.

Future studies should consider analysing other tissue types as well. In a study by Liu et al. (2025), 22 species were caught in the Wanquan River, China, and 12 neonicotinoids were analysed in the liver and muscle tissue. Acetamiprid, clothianidin, dinotefuran, imidacloprid and thiamethoxam were detected in both the fish liver and muscle tissues; however, 10 times lower concentrations were found in the muscle than in the liver. A study by Iturburu et al. (2017) investigated the distribution of imidacloprid in different tissues in *Australoheros facetus* (cichlid). The fish were exposed to different concentrations and after 48 h of exposure, approximately 2,500 pg/g of the 2,500 µg/L was found within the brain, and 3,500 pg/g in the liver and gut. In the muscle tissue, only 800 pg/g imidacloprid was detected, indicating that the muscle tissue might not represent real pesticide concentrations in fish since they may metabolise it quicker than it can accumulate.

## Conclusion

This study highlights the occurrence of legacy and emerging pesticides in freshwater fish from the Inkomati and Letaba catchments in southern Africa. Although only *p,p’*-DDE and *p,p’*-DDD were detected among the 19 targeted OCPs, their presence reflects the persistent environmental legacy of DDT use, particularly for malaria vector control. Concentrations were generally low and comparable to international studies; however, their detection in fish tissue confirms long-term environmental persistence. Importantly, this study provides the first report of neonicotinoid residues in wild freshwater fish in southern Africa. Acetamiprid, clothianidin, dinotefuran, imidacloprid, and thiamethoxam were found in fish muscle at low concentrations. Although neonicotinoids are used extensively in South Africa, according to pesticide use maps, this was not reflected in the concentrations measured in fish muscle tissue. Consequently, the low concentrations observed may be interpreted as evidence of low environmental occurrence or low bioaccumulation within fish muscle tissue. Given the limited regional dataset currently available for neonicotinoids, further studies incorporating multiple tissues, together with targeted analysis of water and sediment, are needed to characterise environmental exposure and bioaccumulation. The results highlight key data gaps for contaminants of emerging concern in African freshwater ecosystems and reinforce the need for expanded and sustained monitoring of neonicotinoids and OCPs in southern African freshwater ecosystems. Future studies should include liver and high-lipid tissues to ensure that the low detection of these pesticides is truly due to low exposure concentrations. This work contributes to the foundational data needed for informed environmental management and regulatory decision-making in Africa. Continued generation of high-quality occurrence data will therefore be essential for improving understanding of pesticide distribution, bioaccumulation and ecological risk and for supporting more effective environmental management.

## Supplementary Information

Below is the link to the electronic supplementary material.

Supplementary Material 1.

## Data Availability

The datasets generated during and/or analysed during the current study are available from the corresponding author on reasonable request.
