# Supplementary material for: Legacy (Organochlorine) and Current Use (Neonicotinoid) Pesticides in Fish from Two Subtropical River Basins
Source: Arch Environ Contam Toxicol. 2026 Oct 26;91(3):27. doi: 10.1007/s00244-026-01219-z (PMC13710463; doi:10.1007/s00244-026-01219-z)
Supplement: Supplementary file 1 [file 244_2026_1219_MOESM1_ESM.docx]

Legacy (Organochlorine) and Current Use (Neonicotinoid) Pesticides in Fish from Two Subtropical River Basins.

Marelize Marsay^1^, Chinemerem R. Ohoro^2^, Collins Nimako^3^, Yared B. Yohannes^3^, Yoshinori Ikenaka^1,3^, Nico J. Smit^1^, Mayumi Ishizuka^3^, Victor Wepener^1^

^1^ Water Research Group, Unit for Environmental Sciences and Management, North-West University, Potchefstroom, South Africa.

^2^Department of Environmental Science, School of Ecological and Human Sustainability, College of Agriculture and Environmental Sciences, University of South Africa, Gauteng, South Africa

^3^Laboratory of Toxicology, Department of Environmental Veterinary Sciences, Faculty of Veterinary Medicine, Hokkaido University, Sapporo, Japan

Corresponding author: 24279862@nwu.ac.za

**Online Resources**

Table S1: Regression equations, correlation coefficients and the limit of detection (LOD) and quantification (LOQ) of OCPs analysis of the Letaba catchment samples.

| Analyte | Regression equation | Correlation Coefficient | LOD ng/g | LOQ ng/g |
| --- | --- | --- | --- | --- |
| α-BHC | Y = 1.423x-0.192 | 0.9997 | 139 | 418 |
| Lindane | Y = 1.179x-0.038 | 1.0000 | 59 | 178 |
| δ-BHC | Y = 0.961x-0.143 | 0.9994 | 141 | 423 |
| Hexochlorobenzene | Y = 0.985x0.066 | 0.9994 | 105 | 314 |
| Heptachlor | Y = 0.773x+0.011 | 0.9994 | 428 | 1285 |
| Heptachlor-exo-epoxide | Y = 0.262x+0016 | 0.9999 | 353 | 1058 |
| Oxy-Chlordane | Y = 0.321x+0.028 | 0.9995 | 498 | 1494 |
| Trans-Chlordane | Y = 0.497x+0.027 | 1.0000 | 135 | 404 |
| Cis-Chlordane | Y = 0.457x+0.044 | 0.9997 | 291 | 872 |
| Trans-Nonachlor | Y = 0.361x+0.024 | 0.9999 | 262 | 786 |
| Cis-Nonachlor | Y = 0.319x+0.021 | 0.9999 | 339 | 1017 |
| Aldrin | Y = 0.282x+0.011 | 0.9991 | 261 | 782 |
| Endrin | Y = 0.119x+0.050 | 0.9927 | 2990 | 8970 |
| Dieldrin | Y = 0.183x-0.087 | 0.9964 | 1234 | 3702 |
| *o,p’*-DDE | Y = 3.074x+0.020 | 0.991 | 31 | 94 |
| *p,p’*-DDE | Y = 2.067x+0.221 | 0.9997 | 40 | 119 |
| *o,p’*-DDD | Y = 4.087x+0.207 | 0.9999 | 32 | 97 |
| *p,p’*-DDD and *o,p’*-DDT | Y = 6.433x-0.093 | 0.9998 | 11 | 33 |
| *p,p’*-DDT | Y = 1.844x-0.586 | 0.9990 | 564 | 1693 |

Table S2: Regression equations, correlation coefficients and the limit of detection (LOD) and quantification (LOQ) of OCPs analysis of the Inkomati catchment samples.

| Analyte | Regression equation | Correlation Coefficient | LOD ng/g | LOQ ng/g |
| --- | --- | --- | --- | --- |
| α-BHC | Y = 1.513x-0.010 | 0.9998 | 12 | 37 |
| Lindane | Y = 1.337x+0.010 | 0.9992 | 23 | 68 |
| δ-BHC | Y = 0.967x-0.349 | 0.9969 | 48 | 145 |
| Hexochlorobenzene | Y = 1.133x+0.128 | 0.9995 | 21 | 62 |
| Heptachlor | Y = 0.912x-0.137 | 0.9994 | 25 | 76 |
| Heptachlor-exo-epoxide | Y = 0.283x+0.014 | 0.9999 | 76 | 229 |
| Oxy-Chlordane | Y = 0.354x+0.002 | 0.9998 | 64 | 192 |
| Trans-Chlordane | Y = 0.532x-0.039 | 0.9995 | 46 | 138 |
| Cis-Chlordane | Y = 0.509x-0.033 | 0.9997 | 52 | 156 |
| Trans-Nonachlor | Y = 0.379x-0.021 | 0.9998 | 57 | 170 |
| Cis-Nonachlor | Y = 0.332x-0.023 | 0.9995 | 81 | 243 |
| Aldrin | Y = 0.321x-0.005 | 0.9990 | 696 | 2087 |
| Endrin | Y = 0.162x-0.018 | 0.9998 | 88 | 265 |
| Dieldrin | Y = 0.162x-0.016 | 0.9989 | 213 | 639 |
| *o,p’*-DDE | Y = 2.780x+0.059 | 0.9998 | 4 | 13 |
| *p,p’*-DDE | Y = 2.046x+0.073 | 0.9999 | 5 | 16 |
| *o,p’*-DDD | Y = 4.008x+0.386 | 0.9984 | 1 | 3 |
| *p,p’*-DDD and *o,p’*-DDT | Y = 6.308x-0.951 | 0.9992 | 5 | 15 |
| *p,p’*-DDT | Y = 2.132x-0.600 | 0.9988 | 25 | 76 |

Table S3: Recovery (%) of OCPs using the appropriate extraction protocol for tissue

| Analytes | Recovery |
| --- | --- |
| α-BHC | 82 |
| Lindane | 97 |
| δ-BHC | 152 |
| Hexochlorobenzene | 73 |
| Heptachlor | 72 |
| Heptachlor-exo-epoxide | 102 |
| Oxy-Chlordane | 89 |
| Trans-Chlordane | 77 |
| Cis-Chlordane | 101 |
| Trans-Nonachlor | 100 |
| Cis-Nonachlor | 140 |
| Aldrin | 102 |
| Endrin | 123 |
| Dieldrin | 84 |
| *o,p’*-DDE | 77 |
| *p,p’*-DDE | 95 |
| *o,p’*-DDD | 127 |
| *p,p’*-DDD and *o,p’*-DDT | 138 |
| *p,p’*-DDT | 117 |

Table S4: Regression equations, correlation coefficients and the limit of detection (LOD) and quantification (LOQ) for neonicotinoids tested.

| Analyte | Regression equation | Correlation Coefficient | LOD pg/g | LOQ pg/g |
| --- | --- | --- | --- | --- |
| 4OH-Imidacloprid | Y = 0.913x+0.007 | 0.999 | 146 | 453 |
| 5OH-Imidacloprid | Y = 0.765x+0.020 | 0.994 | 374 | 1169 |
| Acetamiprid | Y = 41.781x-0.124 | 0.999 | 2 | 5 |
| Clothianidin | Y = 0.983x+0.009 | 0.998 | 153 | 478 |
| Dinotefuran | Y = 1.029x+0.005 | 0.999 | 36 | 117 |
| dm-Acetamiprid | Y = 0.722x-0.011 | 0.994 | 421 | 1235 |
| dm-Clothianidin | Y = 1.013x+0.002 | 0.999 | 61 | 187 |
| Imidacloprid | Y = 1.147x-0.011 | 0.998 | 113 | 416 |
| Nitenpyramid | Y = 0.681x+0.018 | 0.998 | 244 | 781 |
| Sulfoxaflor | Y = 0.805x-0.003 | 0.998 | 198 | 587 |
| Thiacloprid | Y = 0.977x+0.028 | 0.996 | 236 | 762 |
| Thiamethoxam | Y = 11.021x+0.108 | 0.995 | 17 | 70 |

Table S5: Recovery (%) of neonicotinoids using the appropriate extraction protocol for tissue

|  | 1 ppb | | 10 ppb | |
| --- | --- | --- | --- | --- |
| Analytes | Concentration | Recovery | Concentration | Recovery |
| 4OH-Imidacloprid | 0.24 ± 0.43 | 24 | 4.85 ± 0.50 | 49 |
| 5OH-Imidacloprid | 0.15 ± 0.57 | 15 | 5.03 ± 0.33 | 50 |
| Acetamiprid | 0.90 ± 0.03 | 90 | 4.94 ± 0.30 | 49 |
| Clothianidin | 0.76 ± 0.05 | 75 | 5.24 ± 1.86 | 52 |
| Dinotefuran | 0.90 ± 0.06 | 90 | 5.12 ± 0.19 | 52 |
| dm-Acetamiprid | 1.25 ± 0.04 | 125 | 6.24 ± 0.27 | 62 |
| dm-Clothianidin | 0.90 ± 0.04 | 90 | 4.87 ± 0.10 | 49 |
| Imidacloprid | 0.94 ± 0.05 | 94 | 5.23 ± 0.94 | 52 |
| Nitenpyramid | 0.76 ± 0.03 | 76 | 5.82 ± 0.23 | 58 |
| Sulfoxaflor | 0.92 ± 0.08 | 92 | 5.02 ± 0.13 | 50 |
| Thiacloprid | 0.46 ± 0.04 | 46 | 3.7 ± 0.04 | 37 |
| Thiamethoxam | 0.73 ± 0.05 | 73 | 4.74 ± 0.49 | 47 |

Table S6: Water quality variables measured in Tzaneen Dam, Letaba, Sabie, Crocodile and Inkomati rivers. Temp – temperature, EC – electrical conductivity, DO – dissolved oxygen

|  | **pH** | **Temp (^o^C)** | **EC (µS/cm)** | **DO (mg/L)** |
| --- | --- | --- | --- | --- |
| Tzaneen Dam | 7.8 ± 0.38 | 22.1 ± 4.00 | 58 ± 27.4 | 9.2 ± 0.77 |
| Letaba River | 7.9 ± 0.15 | 22.4 ± 1.35 | 73 ± 3.20 | 7.2 ± 0.15 |
| Sabie River | 7.5 ± 0.09 | 20.7 ± 0.10 | 161 ± 1.29 | 7.4 ± 1.29 |
| Crocodile River | 8.1 ± 0.01 | 21.2 ± 0.20 | 460 ± 2.52 | 6.9 ± 0.73 |
| Inkomati River | 8.6 ± 0.14 | 21.7 ± 0.32 | 1162 ± 32.3 | 9.7 ± 0.15 |

Table S7: Mean (± SD) body length (mm) and body weight (g) of fish species collected from the Letaba and Inkomati catchments. Values are presented by species and sampling location, with *n* representing the number of individuals analysed. NA – Not available.

|  |  | **Length (mm)** | **Weight (g)** | **n** |
| --- | --- | --- | --- | --- |
| *Anguilla marmorata* | Inkomati | 875 ± 169 | 1463 ± 1003 | 12 |
| *Anguilla mossambica* | Inkomati | 616 ± 99 | 490 ± 240 | 7 |
| *Coptodon rendalli* | Letaba | NA | NA | 5 |
| *Clarias gariepinus* | Crocodile | 490.0 ± 15.8 | 777.5 ± 42.2 | 4 |
|  | Sabie | 596.7 ± 254.6 | 840.0 ± 272.9 | 4 |
|  | Inkomati | NA | NA | 4 |
|  | Tzaneen Dam | 810.5 ± 83.5 | 3765.0 ± 1405.0 | 3 |
| *Hydrocynus vittatus* | Crocodile | 461.0 ± 76.3 | 1415.0 ± 371.1 | 10 |
|  | Sabie | 327.5 ± 27.5 | 225.0 ± 25.0 | 5 |
|  | Inkomati | NA | NA | 4 |
| *Labeobarbus marequensis* | Letaba | 123.7 ± 25.7 | 16.8 ± 11.8 | 17 |
| *Labeo cylindricus* | Letaba | 149 | 28.6 | 1 |
| *Marcusenius krameri* | Letaba | 156.0 ± 13.0 | 27.9 ± 8.3 | 2 |
|  | Tzaneen Dam | 165.0 ± 8.2 | 35.9 ± 3.8 | 3 |
| *Schilbe depressirostris* | Tzaneen Dam | 180 | 43.9 | 1 |

Length and weight data are unavailable for a subset of the collected fish specimens because these individuals were collected as part of a biodiversity survey, during which morphometric measurements were not recorded in the field. Consequently, these data could not be included in the present study.

Figure S1: *p,p’*-DDD concentrations (ng/g dry weight, mean ± SEM), (A) Clothianidin, (B) Dinotefuran and (C) Thiamethoxam, measured in the muscle tissue of fish species collected from the (A) Letaba catchment (Tzaneen Dam and Letaba River) and (B) Inkomati catchment (Crocodile and Inkomati rivers).

Figure S2: *p,p’*-DDE concentrations (ng/g dry weight, mean ± SEM), (A) Clothianidin, (B) Dinotefuran and (C) Thiamethoxam, measured in the muscle tissue of fish species collected from the (A) Letaba catchment (Tzaneen Dam and Letaba River) and (B) Inkomati catchment (Crocodile and Inkomati rivers).

Figure S3: Acetamiprid concentrations (pg/g dry weight, mean ± SEM) measured in the muscle tissue of fish species collected from the (A) Letaba catchment (Tzaneen Dam and Letaba River) and (B) Inkomati catchment (Sabie, Crocodile and Inkomati rivers).

Figure S4: Imidacloprid concentrations (pg/g dry weight, mean ± SEM) measured in the muscle tissue of fish species collected from the Inkomati catchment (Sabie, Crocodile and Inkomati rivers).

Figure S5: Neonicotinoid concentrations (pg/g dry weight, mean ± SEM), (A) Clothianidin, (B) Dinotefuran and (C) Thiamethoxam, measured in the muscle tissue of fish species collected from the Inkomati River.
